# Factors affecting extubation time following pediatric ambulatory surgery: an analysis using electronic anesthesia records from an academic university hospital

**DOI:** 10.1186/s40981-017-0108-3

**Published:** 2017-07-26

**Authors:** Akihiro Kanaya, Norifumi Kuratani, Yoshinori Nakata, Masanori Yamauchi

**Affiliations:** 1 Department of Anesthesiology and Perioperative Medicine, Tohoku University School of Medicine, 1-1, Seiryo-machi, Aoba-ku, Sendai, 980-8574 Japan; 2 Department of Anesthesia, Saitama Children’s Medical Center, Saitama, Japan; 3 Department of Anesthesia, Teikyo University Hospital, Teikyo University Graduate School of Public Health, Tokyo, Japan

**Keywords:** Emergence, Pediatric ambulatory surgery, Hypercapnia, Hypothermia

## Abstract

**Background:**

In pediatric general anesthesia, our goal should be quicker extubation to facilitate rapid turnover in the operating room without compromising on safety and quality of anesthesia. Although many studies have focused on improving safety and pursuing a higher quality of recovery, factors related to anesthesia emergence remain unclear. We must, therefore, identify factors that influence the process of emergence from general anesthesia in children.

**Findings:**

We retrospectively examined 148 children (aged 1–6 years, American Society of Anesthesiologists physical status: 1–2) who had undergone <2 h of ambulatory surgery. Clinical measures included time from the end of surgery to extubation (extubation time), age, height, weight, surgical time, mean indirect blood pressure during surgery, mean heart rate during surgery, mean end-tidal carbon dioxide during surgery (mETCO_2_), mean body temperature during surgery (mBT), and total amount of fentanyl. Anesthetic procedures involved sevoflurane or propofol. Multiple regression analysis revealed that mETCO_2_ (*p* < 0.01) and mBT (*p* < 0.01) were independent clinical factors associated with extubation time following pediatric ambulatory surgery.

**Conclusions:**

This study of 148 pediatric patients demonstrated that anesthesia emergence may be associated with mBT and mETCO_2_ following pediatric ambulatory surgery. These results show that perioperative vital signs are important in the prevention of delayed emergence for pediatric patients.

## Findings

### Introduction

During recovery from general anesthesia, pediatric patients are more likely to have respiratory events such as laryngeal and bronchial spasms [1, 2] as well as hypoxemia due to their immature alveoli, increased dead space, and increased metabolic rate in comparison with adult patients. Furthermore, unpredicted delayed emergence from general anesthesia can occur in pediatric patients. Safe and faster emergence is crucial in pediatric general anesthesia. In pediatric ambulatory surgical suites, the goal should be faster extubation to facilitate rapid turnover in the operating room without compromising on safety and quality of anesthetic care.

Although many studies have focused on improving safety and pursuing a higher quality of recovery [3–5], factors related to quicker extubation remain unclear. Time from the end of surgery to tracheal extubation (extubation time) is one of the indices of the time from emergence from general anesthesia [6]. We have previously reported on the possible association between extubation time and intraoperative hypercapnia in pediatric general anesthesia [6]. However, the effects of other confounding variables, including vital signs and anesthesia management, were not included in previous analyses. Evidently, because anesthesia emergence is defined as total recovery of central nervous system function, no single factor would play a pivotal role in anesthesia recovery. Here, we analyzed data from an electronic anesthesia retrieval system to identify independent factors associated with tracheal extubation time in pediatric ambulatory patients.

## Methods

After obtaining approval from the institutional review board at Tohoku University Hospital, we conducted a retrospective cohort study. We used an electronic anesthesia database (PrimeGaia, Nihon Kohden Co, Tokyo, Japan) to identify all children aged 1–6 years who had undergone <2 h of ambulatory surgery (minor urologic, skin, and eye surgeries) without postoperative radiographic confirmation and postoperative procedures at our hospital from January 2014 to April 2016. Exclusion criteria included American Society of Anesthesiologists physical status >2 or having undergone mask ventilation without endotracheal intubation during surgery and having been extubated under general anesthesia.

Anesthesia management, including ventilator management, was performed under the direction of board-certified attending anesthesiologists assigned to the respective cases. Generally, anesthesia was maintained with propofol or sevoflurane. Blood pressure was measured at 2.5-min intervals. Heart rate, oxygen saturation, body temperature (oral temperature), and end-tidal carbon dioxide saturation were continuously measured and extracted. We obtained the following information from the electronic anesthesia database: age, height, weight, surgical time, time from the end of surgery to extubation (extubation time), and total amounts of fentanyl. We hypothesized that time to tracheal extubation could be associated with the time of anesthesia emergence because all patients underwent controlled ventilation and were extubated when they were awake. In addition, the completion of surgery and discontinuation of anesthetic administration were almost done at the same time.

Continuous data are expressed as mean ± standard deviation (SD). The mean of all end-tidal carbon dioxide values measured at 3-s intervals during general anesthesia (mETCO_2_) was calculated. We investigated clinical factors associated with the extubation time after the end of pediatric ambulatory surgery using multiple regression analysis. In our preliminary data, patients in the hypercapnia group (*n* = 125; mETCO_2_ ≥ 40 mmHg) were extubated significantly faster (9.8 ± 8.4 min) than those in the hypocapnia group (*n* = 125; mETCO_2_ < 40 mmHg; 16.4 ± 8.0 min) [6]. Based on an estimated 5-min difference in emergence time between the hypercapnia and hypocapnia groups, it was determined that a sample size of ≥41 patients would be required in each group to demonstrate a significant difference at the 0.05 level (power = 0.8; SD = 8 min). All statistical analyses were performed using R version 3.0.1 (R Foundation for Statistical Computing, Vienna, Austria), and *p* values <0.05 were considered statistically significant.

## Results

Our analysis included 148 patients; demographic and clinical data are shown in Table 1. A multiple regression analysis was performed to adjust for age, anesthesia method, total amount of fentanyl, surgical time, mean body temperature (mBT), mean heart rate, mean blood pressure, and mETCO_2_ (Table 2). Results revealed that mETCO_2_ (*p* < 0.01) and mBT (*p* < 0.01) were independent clinical factors associated with tracheal extubation time following pediatric ambulatory surgery.

**Table 1:** Patients’ demographic and operative data

Age (years)	2.9 ± 1.7
Height (cm)	92.2 ± 15.7
Body weight (kg)	14.0 ± 3.9
Operation time (min)	57.1 ± 26.7
mBP (mmHg)	61.9 ± 9.8
mHR (bpm)	101.5 ± 18.1
mETCO_2_ (mmHg)	43.6 ± 7.8
mBT (°C)	37.2 ± 0.7
Fentanyl (mcg/kg)	4.1 ± 3.7
Anesthesia (sev/propo)	111/37

Data are expressed as mean ± standard deviation. *mBP* mean indirect blood pressure during surgery, *mHR* mean heart rate during surgery, *mETCO2* mean end-tidal carbon dioxide during surgery, *mBT* mean body temperature during surgery, *sev/propo* sevoflurane anesthesia/propofol anesthesia

**Table 2:** Results of multiple regression analysis for clinical factors related to time to tracheal extubation

	Estimate	Std. error	*t* value	Pr(>|*t*|)
(Intercept)	165.651	37.921	4.368	<0.000
Age (years)	0.285	0.419	0.679	0.498
Operation time (min)	−0.020	0.025	−0.828	0.409
fentanyl (mcg/kg)	0.175	0.173	1.012	0.314
mBT (°C)	−3.463	1.033	−3.351	0.001
mHR (bpm)	−0.043	0.040	−1.082	0.281
mBP (mmHg)	−0.036	0.060	−0.604	0.547
mETCO_2_ (mmHg)	−0.420	0.092	−4.583	<0.000
Anesthesia (sev vs propo)	1.770	1.645	1.076	0.284

Sev vs. propo, sevoflurane anesthesia vs. propofol anesthesia

## Discussion

Our results suggest that delayed anesthesia emergence may be prevented by avoiding lower mBT and mETCO_2_ in pediatric patients. Importantly, the method of anesthetic management and total amounts of fentanyl are not factors associated with tracheal extubation time. Findings suggest that perioperative vital signs are very important in avoiding delayed emergence for pediatric patients.

Some previous studies [7–9] suggested that hypercapnia-induced hyperpnea shortens the emergence time from inhalation anesthetics. Hypercapnia dilates the cerebral arterial smooth muscle and increases blood flow. The increase in blood flow facilitates rapid elimination of inhaled anesthetics from the brain, and hyperventilation decreases the arterial concentration of the inhaled anesthetic [10]. In addition, our previous research showed that intraoperative hypercapnia may facilitate faster tracheal extubation after ambulatory surgery in pediatric patients [6]. Hypercapnia causes carbon dioxide diffusion into the cerebrospinal fluid from the cerebral vessels and liberates H^+^ ions that stimulate the central chemoreceptors lying below the ventral surface of the medulla. Consequently, hypercapnia might accelerate spontaneous breathing and emergence after surgery. Conversely, it has been suggested that perioperative hypocapnia is associated with the risk of delayed anesthesia emergence and poor cognitive function in adult patients [11]. The findings of this study, which indicate that lower mETCO_2_ may increase emergence time in pediatric ambulatory surgery, are consistent with the results from these studies.

The detailed mechanisms of faster tracheal extubation associated with higher body temperature remain unknown, but it is possible that the effects of anesthetics are prolonged by decreased hepatic flow, hypometabolic state, and decreased renal blood flow and clearance in hypothermia. Previous studies [12, 13] have suggested that hypothermia leads to late emergence, and our finding that delayed emergence is associated with lower body temperature is consistent with these previous reports.

Previous review article [14] suggested that several risk factors, including hyperventilation and hypothermia, delay anesthesia emergence. However, the most important risk factors in pediatric patients remained unknown. Therefore, the findings of our study, using clinical data that lower mBT and mETCO_2_, among several clinical factors, that were associated with delayed anesthesia emergence in pediatric ambulatory surgery has important significance.

This study has several limitations. First, the study was retrospective and original measures were not primarily designed to discern independent factors associated with extubation time following pediatric surgery. Although we used multiple regression analysis to identify several confounding factors, not all relevant factors were extracted. In addition, the method of anesthesia and administration of drugs (e.g., analgesic agents and muscle relaxants) differed across attending anesthesiologists. However, the criteria for extubation (namely, that patients have regular spontaneous breathing and are awake and responsive with stable vital signs) were almost uniform in our hospital. Second, this study had a small sample size, although 148 subjects was sufficient based on our power analysis. Third, the safety of perioperative hypercapnia in children is unclear; however, no remarkable complications arose in our study. Fourth, we needed to include several different types of ambulatory surgery to increase the sample size.

To conclude, this study of 148 pediatric patients demonstrated that anesthesia emergence may be associated with mBT and mETCO_2_ following pediatric ambulatory surgery. We emphasize a close relationship between perioperative vital signs and anesthesia emergence in pediatric ambulatory surgery. Anesthesiologists should carefully monitor patients to avoid perioperative lower mBT and mETCO_2_ to prevent delayed emergence in pediatric patients.

## References

[1] Mc DC (2013). Interventions guided by analysis of quality indicators decrease the frequency of laryngospasm during pediatric anesthesia. Paediatr Anaesth.

[2] Orestes MI, Lander L, Verghese S, Shah RK (2012). Incidence of laryngospasm and bronchospasm in pediatric adenotonsillectomy. Laryngoscope.

[3] Dahmani S, Mantz J, Veyckemans F (2012). Case scenario: severe emergence agitation after myringotomy in a 3-yr-old child. Anesthesiology.

[4] Kanaya A (2016). Emergence agitation in children: risk factors, prevention, and treatment. J Anesth.

[5] Kanaya A, Kuratani N, Satoh D, Kurosawa S (2014). Lower incidence of emergence agitation in children after propofol anesthesia compared with sevoflurane: a meta-analysis of randomized controlled trials. J Anesth.

[6] Kanaya A, Kuratani N, Kaiho Y, Yamauchi M (2016). Intraoperative hypercapnia and anesthesia emergence after pediatric ambulatory surgery. Paediatr Anaesth.

[7] Gopalakrishnan NA, Sakata DJ, Orr JA, McJames S, Westenskow DR (2007). Hypercapnia shortens emergence time from inhaled anesthesia in pigs. Anesth Analg.

[8] Katznelson R, Djaiani G, Naughton F, Wasowicz M, Ragoonanan T, Duffin J, Fedorko L, Murphy J, Fisher JA (2013). Post-operative hypercapnia-induced hyperpnea accelerates recovery from sevoflurane anesthesia: a prospective randomised controlled trial. Acta Anaesthesiol Scand.

[9] Sakata DJ, Gopalakrishnan NA, Orr JA, White JL, Westenskow DR (2007). Rapid recovery from sevoflurane and desflurane with hypercapnia and hyperventilation. Anesth Analg.

[10] Eger EI, Saidman LJ (2005). Illustrations of inhaled anesthetic uptake, including intertissue diffusion to and from fat. Anesth Analg.

[11] Curley G, Kavanagh BP, Laffey JG (2011). Hypocapnia and the injured brain: evidence for harm. Crit Care Med.

[12] Tekgul ZT, Pektas S, Yildirim U, Karaman Y, Cakmak M, Ozkarakas H, Gonullu M (2015). A prospective randomized double-blind study on the effects of the temperature of irrigation solutions on thermoregulation and postoperative complications in percutaneous nephrolithotomy. J Anesth.

[13] Tanaka M, Nagasaki G, Nishikawa T (2001). Moderate hypothermia depresses arterial baroreflex control of heart rate during, and delays its recovery after, general anesthesia in humans. Anesthesiology.

[14] Frost EM (2014). Differential diagnosis of delayed awakening from general anesthesia: a review. Middle East J Anaesthesiol.

